# Characterization of the complete chloroplast genome of *Fibraurea recisa* Pierre 1885 (Menispermaceae), an important medicinal herb from Yunnan, China

**DOI:** 10.1080/23802359.2022.2051760

**Published:** 2022-03-15

**Authors:** Lan-Ping Zheng, Rui Feng

**Affiliations:** College of Chinese Materia Medica and Yunnan Key Laboratory of Southern Medicinal Utilization, Yunnan University of Chinese Medicine, Kunming, Yunnan, China

**Keywords:** Illumina sequencing, phylogenetic analysis, Menispermaceae

## Abstract

*Fibraurea recisa* Pierre 1885, which belongs to the family Menispermaceae, is an important medicinal herb in Yunnan, China. Despite its importance, genetic studies on this species remain rare. Therefore, in the current study, we assembled the complete chloroplast genome of *F. recisa*. Results showed that the complete genome was 161,671 bp in length, including a large single-copy region (LSC, 91,071 bp), small single-copy region (SSC, 20,858 bp), and two inverted repeat regions (IRa and IRb, 24,871 bp). The genome consisted of 124 genes, including 87 protein-coding genes, 29 tRNA genes, and eight rRNA genes. Phylogenetic analysis indicated that *F. recisa* was most closely related to species of *Tinospora* within Menispermaceae. Our complete chloroplast genome of *F. recisa* should contribute to the genetic resource assessment of this species as well as its future conservation and utilization.

*Fibraurea recisa* Pierre 1885 is a member of the family Menispermaceae and is an important medicinal herb in Yunnan, China (Gao et al. 2005). The active ingredient in *F. recisa* exhibits extensive anti-inflammatory and antibacterial effects (Zhu 2006). Thus far, studies on *F. recisa* have focused on the pharmacology and isolation of chemical compounds, while molecular studies remain scarce (Zhu 2006; Wang et al. 2014). Therefore, to promote the conservation and utilization of the genetic resources of this species, we assembled the first complete chloroplast genome of *F. recisa*.

We sampled several *F. recisa* plants from Pingbian, Yunnan, China (23°01′55″ N, 103°44′53″ E), and deposited a voucher specimen in Yunnan University of Chinese Medicine (L-P Zheng, casperlp@126.com) (specimen number YUCM2020F004). Total genomic DNA was extracted from fresh leaves using CTAB method, and the purified genomic DNA library was constructed and sequenced using the Illumina NovaSeq 6000 Platform (Benagen Tech Solution Co., Ltd, Wuhan, China). The genome was assembled using SPAdes v3.6.1 (Bankevich et al. 2012) and sequence annotation was conducted with GeSeq (Tillich et al. 2017).

The complete chloroplast genome of *F. recisa* was 161,671 bp in length, including a large single-copy region (LSC, 91,071 bp), small single-copy region (SSC, 20,858 bp), and two inverted repeat regions (IRa and IRb, 24,871 bp). The complete chloroplast genome showed a typical quadripartite structure, consistent with that of most angiosperm chloroplast genomes. The chloroplast genome consisted of 124 genes, including 87 protein-coding genes, 29 tRNA genes, and eight rRNA genes, with a GC content of 37.76%.

To examine the phylogenetic relationships, phylogenetic analysis was performed based on the chloroplast genomes of *F. recisa* and 14 related species (Figure 1). Sequences were aligned using MAFFT v7 (Katoh and Standley 2013), and a maximum-likelihood (ML) tree was constructed using IQ-TREE with 5 000 ultrafast bootstraps (Nguyen et al. 2015). Results showed that *F. recisa* formed a sister clade to the species of *Tinospora*, and formed another sister clade to *Menispermum*, *Sinomenium*, *Pericampylus*, and *Stephania* within Menispermaceae, with 100% support (Figure 1). Thus, our results indicated that *Fibraurea* was most closely related to the species of *Tinospora*, generally consistent with previous studies (Wang et al. 2017). Our complete chloroplast genome of *F. recisa* should contribute to the genetic resource assessment of this species as well as its conservation and utilization in the future.

**Figure 1.**
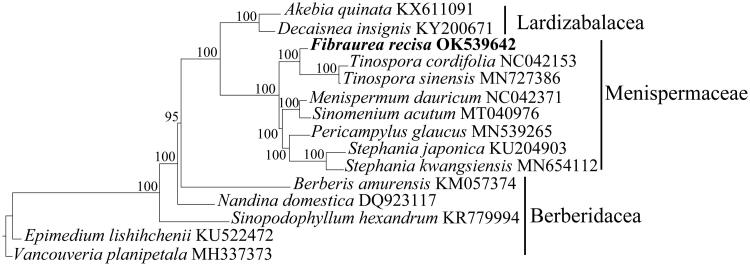
Phylogenetic tree of *Fibraurea recisa* and 14 related species based on complete chloroplast genomes. Nodal numbers are ML bootstrap values.

## Author contributions

ZLP designed the study, ZLP and FR analyzed the data, ZLP prepared and revised the manuscript. All authors have read and agreed to the published version of the manuscript.

## Ethics statement

This study did not require any ethical or institutional approvals according to “Wild Plants Protection Regulations of the People's Republic of China” and “Wild Medicinal Materials Resources Protection Regulations of the People's Republic of China”, because this species is not in the list of the protected plants or medicinal materials of China and the sampling was conducted outside reserves.

## Data Availability

The genome sequence data of this study are openly available in GenBank of NCBI (https://www.ncbi.nlm.nih.gov/) under accession no. OK539642. The associated Bio-Project, Bio-Sample, and SRA numbers are PRJNA748215, SAMN20309845, and SRR15214094, respectively.
